# Editorial: The Integrative Physiology of Metabolic Downstates

**DOI:** 10.3389/fphys.2021.758972

**Published:** 2021-09-13

**Authors:** Alessandro Silvani

**Affiliations:** Department of Biomedical and Neuromotor Sciences, Alma Mater Studiorum Università di Bologna, Bologna, Italy

**Keywords:** hibernation, sleep, torpor, metabolism, physiology

Homeostasis relies upon the exquisite integration of diverse physiological functions, such as neuromuscular and cardiorespiratory functions and energy and thermal balance, in the face of external and internal challenges. The latter include physical exercise, which represents a short-term “metabolic upstate” of increased energy expenditure. To the other end of the spectrum, diverse physiological behaviors including sleep, daily torpor, and hibernation represent “metabolic downstates” of decreased energy expenditure. The study of physical exercise has been key for our current understanding of integrative physiology, for instance highlighting the role of feedforward control by central commands in complementing negative feedback regulation of physiological variables. In contrast, the integrative physiology of “metabolic downstates” remains insufficiently understood.

This Research Topic aimed to contribute to bridge this knowledge gap by bringing together cutting-edge updates on the integrative physiology of “metabolic downstates.” Remarkably, the Research Topic attracted important contributions on the molecular, cellular, and metabolic aspects of the integrative physiology of hibernation. Focusing on molecular mechanisms, Fu et al. reported exciting new evidence on dynamic RNA regulation in the brain of 13-lined ground squirrels (*Ictidomys tridecemlineatus*) in physiologically distinct phases of hibernation, providing evidence for regulated transcription and RNA turnover during hibernation. Wang et al. uncovered hitherto unrecognized dynamics of calcium ions and calcium-handling proteins in the skeletal muscles of hibernating Daurian ground squirrels (*Spermophilus dauricus*), in the face of significantly decreased metabolic activity. Relevant to the cellular aspects of the integrative physiology of metabolic downstates, Huber et al. reported novel evidence of a temperature-compensated reduction of neutrophil oxidative burst capacity in hibernating garden dormice (*Eliomys quercinus*), while Dias et al. reviewed the evidence comparing the molecular aspects of cellular quiescence of hematopoietic stem cells with hibernation at the cellular level, revealing several key shared factors. The general aspects of metabolic adaptation associated with hibernation were reviewed by Giroud et al., who emphasized the mechanisms enabling heterotherms to protect their key organs against potential threats, such as reactive oxygen species. The evidence on protein metabolism in hibernation was reviewed by Bertile et al., focusing on the mechanisms that spare muscle proteins in the face of the inactivity that accompanies hibernation. Lipid metabolism was addressed by Watts et al. who demonstrated that metabolic rate and body mass loss in hibernating garden dormice do not differ with the dietary levels of essential polyunsaturated fatty acids in the fall season preceding hibernation, highlighting a preserved regulation of peroxisome proliferator-activated receptor pathway. Lastly, system-level adaptations to short photoperiod exposure in terms of body mass, fur insulation, locomotor activity, and core body temperature were investigated by Haugg et al. in Djungarian hamsters (*Phodopus sungorus*), providing insights into preconditions and proximate stimuli of hibernation. On an apparently different note, Shirota et al. contributed a detailed report of discrepancies in the time course of sleep stage dynamics, electroencephalographic activity and heart rate variability over sleep cycles in the night of adaptation to the sleep laboratory in healthy young adults. Their study is of interest for the design and interpretation of human sleep studies and is appropriate to this Research Topic because sleep is also a “metabolic downstate” (Silvani et al., 2018).

Taken together, the research and review papers in this Research Topic drafted a remarkable picture of the complexity of the integrative physiology of hibernation, which is the metabolic downstate that was addressed by most contributions. This complexity stems not only from the multiplicity of the levels of physiological integration, but also from the temporal dynamics of hibernation, which is typically interrupted by short inter-bout arousals, and by differences among hibernator species. This complexity also brings about the potential to uncover basic mechanisms that may be conserved in evolution and harnessed to improve healthcare. For instance, understanding the mechanisms that preserve protein mass during hibernation could conceivably help limit muscle protein loss in conditions of inactivity or weight loss therapy. Knowledge of the mechanisms that limit circulating innate immune cell activity in hibernation could have clinical relevance in the context of therapeutic hypothermia and reperfusion injury. In perspective, it is tempting to speculate that greater knowledge of the integrative physiology of hibernation may allow its artificial reproduction in human subjects, with potential implications for healthcare as well as for long-range space exploration (Cerri et al., 2021).

By subtraction, the topics that were less covered in this article collection indicate important areas for future research. One such topic concerns whether and to what extent findings in multi-day hibernating species may be replicated during shallower daily torpor bouts in the house mouse (*Mus musculus*). This replication would allow researchers to leverage the power of genetic manipulations and analyses in Mus musculus, although, as shown by Fu et al. in this article collection, tools for genetic analysis in hibernating mammals are catching up. The other underrepresented topic is integrative sleep physiology, particularly as related to decreases in metabolic rate during sleep. The technical difficulty of measuring multiple physiological variables simultaneously without altering sleep physiology cannot be underestimated, as shown by the distinct physiological effects of habituation to the recording setup reported in this article collection by Shirota et al. even with non-invasive recordings in large animals such as human subjects. Nevertheless, increased knowledge of the integrative physiology of the sleep “metabolic downstate” could also have relevant translational implications, considering the high combined prevalence of sleep disorders and their association with cardiometabolic risk (Silvani, 2019).

## Author Contributions

AS drafted and reviewed the submission.

## Conflict of Interest

The author declares that the research was conducted in the absence of any commercial or financial relationships that could be construed as a potential conflict of interest.

## Publisher's Note

All claims expressed in this article are solely those of the authors and do not necessarily represent those of their affiliated organizations, or those of the publisher, the editors and the reviewers. Any product that may be evaluated in this article, or claim that may be made by its manufacturer, is not guaranteed or endorsed by the publisher.
